# Saliva-Mediated Contrasting Effects of Two Citrus Aphid Species on Asian Citrus Psyllid Feeding Behavior and Plant Jasmonic Acid Pathway

**DOI:** 10.3390/insects14080672

**Published:** 2023-07-28

**Authors:** Jing Gao, Tonglai Tao, Steven P. Arthurs, Mubasher Hussain, Fengxian Ye, Runqian Mao

**Affiliations:** 1 Guangdong Key Laboratory of Animal Conservation and Resource Utilization, Guangdong Public Laboratory of Wild Animal Conservation and Utilization, Guangdong Engineering Research Center for Mineral Oil Pesticides, Institute of Zoology, Guangdong Academy of Sciences, Guangzhou 510260, China; gaoj@giz.gd.cn (J.G.); 15580320297@163.com (T.T.); mubasherhussain05uaf@yahoo.com (M.H.); yefengxian2021@126.com (F.Y.); 2 School of Life Sciences, South China Normal University, Guangzhou 510631, China; 3 Biobee USA, Tucker, GA 30084, USA; steven.arthurs@biobee.us

**Keywords:** citrus aphid, Asian citrus psyllid, saliva, feeding behavior, phytohormone defenses

## Abstract

**Simple Summary:**

The Asian citrus psyllid, *Diaphorina citri* Kuwayama, is one of the most important citrus pests because it transmits the bacterium that causes citrus Huanglongbing during feeding. The saliva of herbivorous insects can modulate plant defenses and, in turn, impact insect fitness, which is mostly studied in insects feeding on herbaceous plants. The role of saliva in the relationship between the Asian citrus psyllid and its woody host plant citrus, on the other hand, is unknown. Because two citrus aphid species, *Aphis spiraecola* Patch and *Aphis* (*Toxoptera*) *citricidus* (Kirkaldy), have a contrasting impact on the performance of subsequently infested *D. citri*, we explored the role of their saliva on *D. citri* feeding behavior and host plant defenses. We found that the infiltrating saliva of *A. spiraecola* into the host citrus leaves of the psyllid disrupted the subsequent feeding behavior of *D. citri* and also activated the expression of genes involved in plant salicylic acid (SA) and jasmonic acid (JA) defense pathways. By contrast, saliva infiltrations of *A. citricidus* (Kirkaldy) promoted *D. citri* feeding, activated the expression of one gene involved in the SA pathway, and repressed several genes involved in the JA pathway. We demonstrate that the saliva of aphids can affect *D. citri* performance, possibly by modulating plant defenses. This is the first study to show that insect saliva can influence *D. citri* feeding behavior by changing plant defenses.

**Abstract:**

While herbivorous insect saliva plays a crucial role in the interaction between plants and insects, its role in the inter-specific interactions between herbivorous insects has received little attention. Pre-infestation of citrus plants with *Aphis spiraecola* Patch and *Aphis* (*Toxoptera*) *citricidus* (Kirkaldy) exhibited positive and negative effects on the performance (feeding and reproduction) of *Diaphorina citri* Kuwayama. We explored the role of saliva in this plant-mediated interaction by infiltrating fresh and boiled aphid saliva into plants and detecting *D. citri* feeding behavior and citrus plant defense response. Leaf infiltration of *A. spiraecola* saliva disrupted the subsequent feeding of *D. citri*, indicated by prolonged extracellular stylet pathway duration and decreased phloem sap ingestion duration. By contrast, infiltration of *A. citricidus* saliva decreased the duration of the extracellular stylet pathway and phloem sap ingestion of *D. citri*. Furthermore, gene expression analysis showed that several salicylic acid (SA)- and jasmonic acid (JA)-pathway-related genes were activated by *A. spiraecola* saliva infiltration. However, two SA-pathway-related genes were activated and three JA-pathway-related genes were suppressed following *A. citricidus* saliva infiltration. Treatment with boiled saliva did not similarly impact *D. citri* feeding behavior or plant defense response. This study suggests that salivary components (those that can be inactivated by heating) from two citrus aphid species differently affect plant defenses and that they were responsible for the contrasting plant-mediated effects of two citrus aphids on the feeding behavior of *D. citri*. This study indicates a novel three-way citrus aphid–plant–citrus psyllid interaction.

## 1. Introduction

Plant-initiated defenses against herbivores may reflect cues from herbivore-associated molecular patterns (HAMPs) present in oral secretions (saliva, regurgitant), oviposition secretions, cuticle, pheromones, or feces [1]. These defenses are herbivore-specific and are commonly regulated by phytohormones, including salicylic acid (SA) and jasmonic acid (JA) (the most characterized oxylipin relevant to plant defense) [2,3,4]. Generally, SA-dependent defenses are induced in response to phloem-feeding insects, while JA-dependent defenses are induced by chewing and some phloem-feeding insects [5,6]. In the evolutionary arms race between insect herbivores and plants, some of the former may suppress plant defense pathways to gain fitness [7]. 

Molecular studies of genes and proteins involved in plant–herbivore interactions suggest that insect saliva are primary HAMPs that trigger plant defenses [8]. Piercing–sucking hemipterans such as aphids and planthoppers use elongated flexible mouthparts (stylets) to deliver saliva and obtain nutrients from phloem sap. During probing and feeding, these insects secrete gelling saliva that forms an intercellular sheath for the stylet and secrete watery saliva to plant mesophyll cells and the vascular system [9,10]. Through the transcriptomic analysis of insect salivary glands and mass spectroscopy analyses of secreted saliva, numerous saliva components have been identified [11,12,13]. Several plant defense elicitors in saliva have been identified. For example, *Myzus persicae* (Sulzer) secrete cysteine protease cathepsin B3 (CathB3) into tobacco plants, which activates a reactive oxygen species defense response and inhibits the feeding of *M. persicae* [14]. The infiltration of *M. persicae* saliva or salivary components (in the range of 3–10 kDa) into *Arabidopsis* plants activates defense signaling and decreases *M. persicae* offspring number [15]. In addition, some salivary components are categorized as ‘effectors’, which can suppress plant defense. For instance, the aphid *Acyrthosiphon pisum* (Harris) secretes a macrophage migration inhibitory factor (MIF) in its saliva, which inhibits the expression of defense-related genes, callose deposition, and hypersensitive cell death, and promotes aphid survival and feeding [16]. Similar findings were reported for whitefly *Bemisia tabaci* (Gennadius) salivary protein ferritin BtFer1, which inhibited JA-dependent signaling, and facilitated whitefly host exploitation [17]. Despite the identification of these salivary proteins, the investigation of the relationship between piercing–sucking insect saliva and plant defense has focused on a few individual model species, such as *M. persicae* [18], *A. pisum* [19], *B. tabaci* [17], and *Nilaparvata lugens* (Stål) [20], which all feed on herbaceous plants. The role of saliva in plant–insect interactions in woody plants has received little attention. 

The Asian citrus psyllid *Diaphorina citri* Kuwayama is a primary pest of citrus due to its transmission of the pathogen causing Huanglongbing, a devastating and uncurable citrus disease [21]. Although transcriptomic and proteomic analyses have analyzed *D. citri* salivary gland gene expression and salivary secretions [13,22,23], the role of saliva in the interaction between *D. citri* and host plants has not been investigated. In citrus groves, *D. citri* are sympatric with other herbivorous insects, including citrus aphids [24]. Similar to *D. citri*, many citrus aphids preferentially feed and develop on young shoots and leaves. We previously reported that the citrus aphids *Aphis spiraecola* Patch and *Aphis* (*Toxoptera*) *citricidus* (Kirkaldy) manipulated citrus phytohormone-dependent defenses in different ways [25] and also that *A. spiraecola* and *A. citricidus* decrease and increase *D. citri* reproduction and feeding, respectively [26]. 

We hypothesized that the saliva of citrus aphids might play a role in the plant-mediated effect of citrus aphids on *D. citri* performance and that saliva from two citrus aphid species may differently affect plant defense responses. In this study, we collected saliva of citrus aphids using an artificial diet; then, saliva was infiltrated into citrus leaves to investigate subsequent effects on *D. citri* feeding and plant defense. The electrical penetration graph (EPG) technique was used to detect *D. citri* feeding behavior, and reverse-transcription quantitative real-time polymerase chain reaction (RT-qPCR) was used to detect the expression of genes involved in SA- and JA-dependent pathways. 

## 2. Materials and Methods

### 2.1. Plants and Insects

Sweet orange (*Citrus sinensis* (L.) Osbeck) grafted on *Citrus tangerine* Tanaka rootstock, obtained from a nursery at Guangxi province, China, was used for experiments. Plants (70–80 cm tall) were planted in 8 L plastic pots and maintained at 25 °C, 16 h light: 8 h dark, and 60–70% humidity. Plants were watered twice a week, fertilized weekly with 1 g/L NPK 20-10-20 (Haidesi Fertilizer Company, Weifang, Shandong, China), and pruned three weeks before experiments to promote flushing.

A laboratory colony of *A. spiraecola* and *A. citricidus*, originally collected from a citrus orchard in Guangzhou, China in 2018, were identified according to species-specific characteristics [27,28]. Parthenogenetic aphids of each species were separately maintained on *C. sinensis*. Plants were covered with an 80-mesh nylon net (40 cm width × 60 cm height) to prevent dispersion. *D. citri* were collected from *Murraya paniculata* (L.) Jack from Sun Yat-sen university at Guangzhou, China in 2019, and maintained on *M. paniculata* (in 8 L plastic pots) in a separate climate-controlled room. Plants and insects were maintained under the same conditions described above.

### 2.2. Aphid Pre-Infestation Treatments

Ten fourth instar *A. spiraecola* or *A. citricidus* were infested on young citrus shoots (4 cm in length) containing flushing (not expanded leaves). Infested and uninfested control shoots were covered with an 80-mesh nylon net (one shoot per plant). Aphids were allowed to feed for 24 h, after which they were removed, and the prior infested and uninfested shoots used to quantify *D. citri* feeding behavior. 

### 2.3. Saliva Collection and Infiltration

Aphid saliva was collected using the double layer membrane method [29]. For each species (*A. spiraecola* and *A. citricidus*), mixed life stages (starved for 12 h) were allowed to feed from 1 mL artificial diet (15% *w*/*v* sucrose) between two layers of Parafilm. The diet was sterilized with a millipore membrane filter (0.22 μm). Parafilm was stretched across a PVC tube (3 cm diameter and 4 cm height), and aphids were transferred into the tubes (150 aphids per tube). Equivalent tubes without aphids served as controls. The tubes were placed in a climate chamber (25 °C, 16 h light: 8 h dark), and the sucrose diet collected 48 h later. Some saliva was boiled at 95 °C for 30 min to inactivate proteins [15]. Sucrose diets containing fresh, boiled, and control saliva samples were infiltrated into young *C. sinensis* leaves using a 1 mL syringe without the needle. For each leaf (2.5 cm in width and 5.5 cm in length), 200 µL diet was infiltrated at the abaxial side [30]. The infiltrated leaves were used for *D. citri* feeding behavior analysis after 24 h. 

### 2.4. Detection of D. citri Feeding Behavior

The effects of both direct aphid infestation and the aphid saliva infiltration on the subsequent feeding behavior of *D. citri* were investigated using an electrical penetration graph (EPG) technique [26]. A gold wire (12.5 µm diameter) was attached to *D. citri* at the pronotum with conductive silver glue, with the other side of the wire connected to the EPG amplifier. Another electrode was inserted into the soil at the base of the tested plant. The *D. citri* was placed on the abaxial side of the leaf, and tested plants were placed in a Faraday cage to prevent external electrical disruption. Only one leaf was used per plant. The stylet activity was recorded continuously for 8 h, with 13 replicates performed on aphid pre-infestation treatments, and 11 replicates performed for each saliva treatment.

Psyllid feeding behaviors were quantified using a 4-channel direct-current EPG system (Giga-4; EPG systems, Wageningen University, The Netherlands) and the signals analyzed by Stylet+ software (stylet +d 1.28/stylet +a 1.30, Wageningen University, The Netherlands). Six waveforms are reported for *D. citri* [31]: non-probing (Np), pooled pathway phase activities (C) (including epidermis first stylet contact, intercellular sheath salivation, and stylet movements), first contact with phloem (D), salivary secretion into sieve elements (E1), phloem sap ingestion (E2), and xylem ingestion (G). We recorded the number of waveform events per insect (NWEI), as well as the mean waveform duration per responding insect (WDI), and the mean waveform duration per event (WDE) (for insects that performed a waveform at least once) of these waveforms [32,33]. 

### 2.5. Expression of Genes Involved in SA and JA Defense Pathways

The saliva collection and infiltration treatments were as described in Section 2.3. The leaves were collected 6 h and 24 h after infiltration and stored at −80 °C until use. Total leaf RNA was extracted using TRIzol reagent (Tiangen, Beijing, China), and 1 μg RNA was used to synthesize the first cDNA strand with FastQuant cDNA (Tiangen, Beijing, China) according to the manufacturer’s instructions. Real-time quantitative-polymerase chain reaction (RT-qPCR) was performed in 20 μL reaction volumes containing 10 μL 2× SYBR Premix (Tiangen, Beijing, China), 8 μL water, 1 μL gene-specific primers, and 1 μL cDNA template. The reaction was performed with a CFX Connect^TM^ Real-Time PCR System (Bio-Rad, Hercules, CA, USA). The temperature protocol was: 95 °C for 30 s, followed by 40 cycles at 95 °C for 10 s, 60 °C for 20 s, and 72 °C for 30 s. The expression of five genes involved in the SA pathway and five genes involved in the JA pathway were detected. *CitGAPC1* was used as an internal control. Sequences and description of primers are listed in Table 1 [34,35,36,37]. There were four replicates per treatment (each leaf was considered a replicate), and each replicate contains three technique replicates.

### 2.6. Statistical Analysis

One-way analysis of variance (ANOVA) and Dunnett’s test was used to compare different EPG variable values, as well as phytohormone-dependent defense-associated gene expression (SPSS 20, SPSS Inc., Chicago, IL, USA). Where necessary to meet assumption of normality and homogeneity of variances, data were log transformed. Results were considered significantly different at *p* < 0.05.

## 3. Results

### 3.1. Effect of Aphid Pre-Infestation on the Feeding Behavior of D. citri

**Figure 1:**
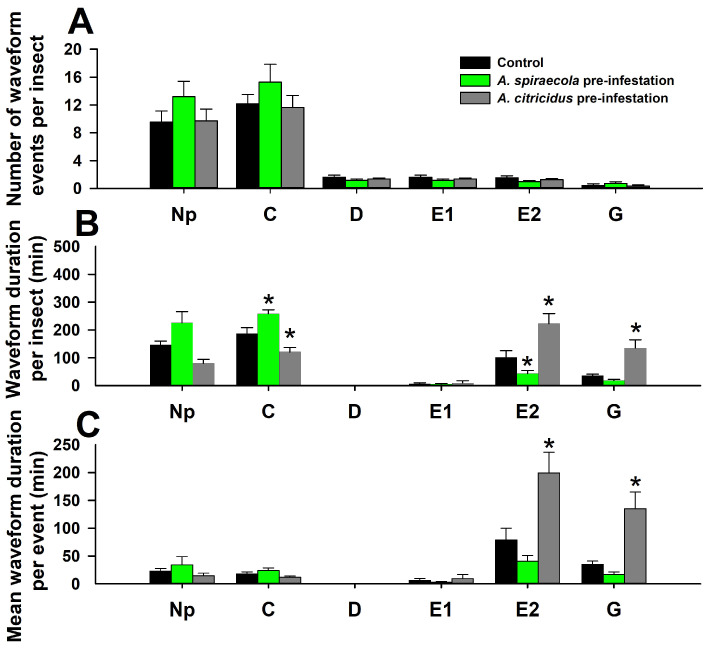
Effect of *A. spiraecola* and *A. citricidus* pre-infestation on the feeding behavior of *D. citri* as determined by EPG. Values show mean ± SE (*n* = 12). (**A**) Number of waveform events per insect; (**B**) waveform duration per insect; (**C**) waveform duration per event. The * indicates significant difference of the treatment when compared with control (ANOVA, Dunnett’s test, *p* < 0.05). Np: non-probing, C: pooled pathway phase activities, D: first contact with phloem, E1: salivary secretion into sieve elements, E2: phloem sap ingestion, G: xylem ingestion.

The EPG variables observed for *D. citri* feeding on *C. sinensis* shoots varied, based on prior aphid infestation. For the number of waveform events per insect (NWEI), none of the six waveforms were significantly affected by *A. spiraecola* or *A. citricidus* pre-infestation when compared with controls (Figure 1A). For the mean waveform duration per insect (WDI), *D. citri* spent a significantly longer time on pathway phase (C), and less time on phloem ingestion (E2) on *A. spiraecola* pre-infested plants. However, pre-infestation by *A. citricidus* decreased the duration of C, and increased the duration of E2 and xylem ingestion (G), when compared with controls. The non-probing (Np), first phloem contact (D), and phloem salivation (E1) were not affected by any aphid pre-infestation when compared with controls (Figure 1B). For the mean waveform duration per event (WDE), only E2 and G were higher on *A. citricidus* pre-infested plants compared with controls. The shoots pre-infested with *A. spiraecola* did not significantly affect any WDE variables of *D. citri* when compared with controls (Figure 1C).

### 3.2. Effect of A. spiraecola and A. citricidus Saliva Infiltration on the Feeding Behavior of D. citri

**Figure 2:**
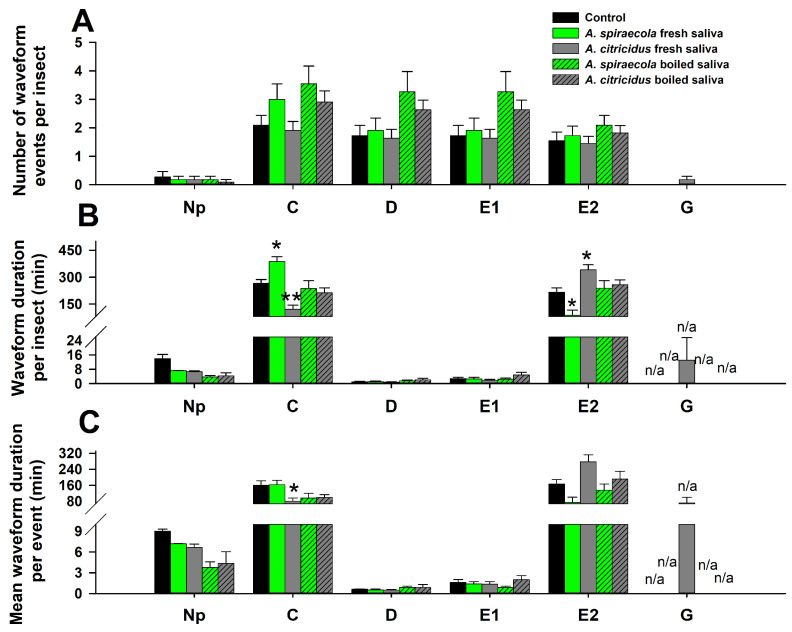
Effect of *A. spiraecola* and *A. citricidus* saliva infiltration on the feeding behavior of *D. citri* as determined by EPG. Values show mean ± SE (*n* = 11). (**A**) Number of waveform events per insect; (**B**) waveform duration per insect; (**C**) waveform duration per event. The asterisk indicates significant difference of the treatment when compared with control (ANOVA, Dunnett’s test, * *p* < 0.05, ** *p* < 0.01). n/a means not statistically analyzed due to low sample numbers.

Some EPG variables for *D. citri* feeding varied based on saliva treatments. For NWEI, none of the six waveforms were significantly affected by any saliva treatment compared with controls (Figure 2A). For WDI, the duration of C was increased, and the duration of E2 was decreased by *A. spiraecola* fresh saliva, compared with controls. By contrast, the duration of C was decreased and the duration of E2 was increased by *A. citricidus* fresh saliva. No significant differences between boiled (*A. spiraecola* or *A. citricidus*) and controls were observed for C and E2 duration. The duration per insect of waveform Np, D, and E1 did not show significant differences between saliva treatments and controls, while G could not be statistically analyzed due to the low sample number (Figure 2B). For WDE, the duration of C was only lower when plants were treated with *A. spiraecola* fresh saliva compared with controls. The duration of G per event also was unable to be statistically analyzed. Other variables associated with WDI did not show significant difference between any saliva treatment and controls (Figure 2C).

### 3.3. Expression of Genes Involved in Phytohormone-Dependent Defense after Citrus Aphid Saliva Infiltration

**Figure 3:**
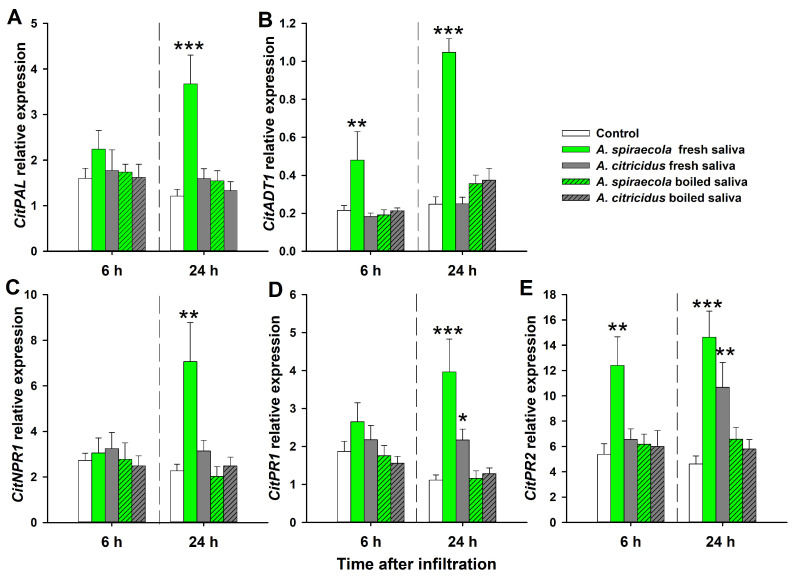
Relative expression of genes involved in salicylic acid (SA) pathway in sweet orange after *A. spiraecola* and *A. citricidus* saliva (fresh and boiled saliva) infiltration. (**A**) Phenylalanine ammonia-lyase (*CitPAL*); (**B**) arogenate dehydratase/prephenate dehydratase 1 chloroplastic 1 (*CitADT1*), (**C**) pathogenesis-related protein 1 (*CitPR1*); (**D**) nonexpresser of pathogenesis-related genes 1 (*CitNPR1*; (**E**) β-1,3-glucanase (*CitPR2*). Values show mean ± SE (*n* = 4). The asterisk indicates significant difference of the treatment when compared with control (ANOVA, Dunnett’s test, * *p* < 0.05, ** *p* < 0.01, *** *p* < 0.001).

All five tested genes involved in the SA pathway were upregulated in *C. sinensis* leaves 24 h after infiltration with fresh *A. spiraecola* saliva compared with controls (Figure 3). Moreover, two of them (*CitADT1* and *CitPR2*) showed significantly increased expression after 6 h (Figure 3B,E). By contrast, exposure of *C. sinensis* leaves to *A. citricidus* fresh saliva only increased the expression of *CitPR1* and *CitPR2* after 24 h (Figure 3D,E). By contrast, exposure of *C. sinensis* leaves to boiled saliva of either aphid species did not result in significant changes in SA pathway gene expression relative to controls. 

**Figure 4:**
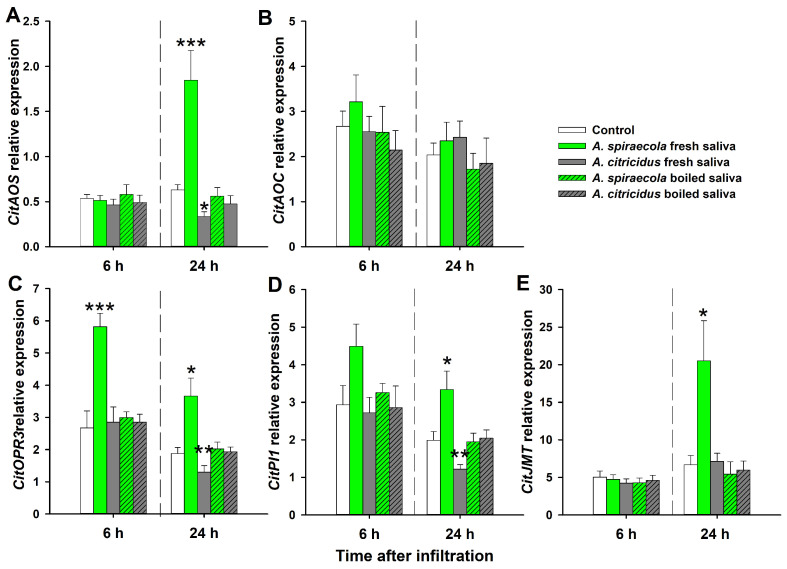
Relative expression of genes involved in jasmonic acid (JA) pathway in sweet orange after *A. spiraecola* and *A. citricidus* saliva (fresh and boiled saliva) infiltration. (**A**) Allene oxide synthase (*CitAOS*); (**B**) allene oxide cyclase (*CitAOC*); (**C**) 12-oxophytodienoate reductase 3 (*CitOPR3*); (**D**) cysteine proteinase inhibitor 1 (*CitPI1*); (**E**) jasmonate methyltransferase (*CitJMT*). Values show mean ± SE (*n* = 4). The asterisk indicates significant difference of the treatment when compared with control (ANOVA, Dunnett’s test, * *p* < 0.05, ** *p* < 0.01, *** *p* < 0.001).

Four of the five tested genes involved in the JA pathway (i.e., *CitAOS*, *CitOPR3*, *CitPI1*, and *CitJMT*) were upregulated in *C. sinensis* leaves 24 h after infiltration with fresh *A. spiraecola* saliva when compared with control (Figure 4). Additionally, *CitOPR3* significantly increased expression after 6 h (Figure 4C). By contrast, infiltration with fresh *A. citricidus* saliva inhibited the expression of three genes involved in the JA pathway (*CitAOS*, *CitOPR3* and *CitPI1*) at 24 h when compared with controls. However, *A. citricidus* fresh saliva did not affect JA pathway gene expression at 6 h after infiltration. In addition, exposure of *C. sinensis* shoots to boiled saliva of either aphid species did not result in significant changes in JA pathway gene expression relative to controls (Figure 4).

## 4. Discussion

Herbivorous insects sharing the same host plant can influence each other through plant-mediated interactions [38], yet in most cases the underlying mechanisms are still unclear. The present study showed that saliva from two different citrus-feeding aphids impacted subsequent feeding behaviors of *D. citri*, which may relate to their different modulation of plant JA-dependent defense. This study provides evidence that saliva mediates the tripartite citrus aphid–plant–citrus psyllid interaction. 

The EPG technique used in this study records the stylet activity and location of piercing–sucking insects in different host plant tissues, with such feeding behavior variables known as indicators of host plant suitability [39,40]. For example, the observation that *A. spiraecola* saliva infiltration resulted in prolonged *D. citri* extracellular stylet pathway duration suggests a stronger mesophyll resistance. Similarly, the shortened duration of phloem sap ingestion by *D. citri* following exposure to *A. spiraecola* saliva is compatible with phloem-located resistance [41]. Such differences indicate that *A. spiraecola* saliva disrupts the feeding behavior of *D. citri* through a plant-mediated interaction, similar to the effect of direct *A. spiraecola* pre-infestation. Saliva-induced feeding changes have been shown in other insects. For example, infiltration of *Sitobion avenae* (F.) saliva into wheat disrupted the feeding of subsequent infested *S. avenae* [29]. By contrast to *A. spiraecola*, different results were observed following *A. citricidus* feeding or saliva infiltration. In this case, the extracellular stylet pathway phase duration was decreased and the phloem ingestion duration was increased, suggesting better feeding efficiency. Although aphid saliva infiltration and aphid feeding caused a similar subsequent effect on *D. citri* feeding, there were some differences. For example, *A. citricidus* feeding increased G of WDI, and E2 and G of WDE, but *A. citricidus* saliva did not. Similarly, while *A. citricidus* saliva decreased C of WDE, the aphid feeding did not. This suggests that the plant–insect interaction is more complex than the saliva–plant interaction, and insect cues other than saliva may also modulate plant defense responses [1]. Proteins are major components of insect saliva [11,42]. In the present study, the differential effects of *A. spiraecola* and *A. citricidus* fresh saliva on *D. citri* feeding were not observed when boiled saliva was used. This finding indicates that aphid salivary proteins may be responsible for the plant-mediated effects on *D. citri*. However, saliva also contains non-protein compounds, such as benzoic acid, phytohormones, and disulfooxy fatty acids [43,44]. Recently, a RNA transcript was shown to be translocated from *M. persicae* to host plants, which promoted aphid fecundity [45]. Treating the saliva with protease before infiltrating it into plants would help confirm the role of salivary proteins.

The EPG data suggest that saliva from two citrus aphid species differently affected plant defense response. To verify this further, we examined saliva infiltration events on phytohormone-dependent defenses. For the genes involved in SA-dependent defense, *A. spiraecola* saliva activated the expression of two genes at 6 h and five genes at 24 h, while *A. citricidus* saliva only activated the expression of two genes at 24 h. For the genes involved in JA-dependent defense, *A. spiraecola* saliva activated the expression of one gene at 6 h and four genes at 24 h, while *A. citricidus* saliva inhibited three genes’ expression at 24 h. This indicates that *A. spiraecola* saliva-induced phytohormone-dependent defense is faster and stronger than that induced by *A. citricidus* saliva. This modulation of plant defense responses by saliva has also been reported in other piercing–sucking insects, including *B. tabaci* [46], *S. avenae* [29], and *M. persicae* [15]. Moreover, the effect of citrus aphid saliva on plant defenses is similar to that induced by citrus aphid infestation, where both aphid species activated genes involved in SA-dependent defense, whereas *A. spiraecola* activated and *A. citricidus* repressed genes involved in JA-dependent defenses [25]. It was previously proposed that plants respond differently to herbivores with different host ranges [47]. In this case, *A. spiraecola* is polyphagous (feeding on *Pyrus*, *Prunus*, *Malus*, and *Citrus* spp.) [48], while *A. citricidus* is oligophagous (feeding on members of Rutaceae) [49]. This degree of host plant specialization could help explain the extent to which saliva plays a role in the co-evolution between plant and herbivorous insects.

The SA- and JA-dependent defense influence plant resistance against herbivores, with JA signaling playing a central role [50]. For example, wheat plants treated with MeJA (methyl jasmonate) significantly increased levels of plant defensive proteins (polyphenol oxidase, peroxidase, and proteinase inhibitor), and disrupted the feeding of *S. avenae* [51]. Foliar application of MeJA on citrus disrupted *D. citri* feeding behavior and reduced its number of offspring [26]. By contrast, the Arabidopsis *coi1* mutant, which has impaired JA defenses, accelerated *B. tabaci* nymph development [52]. Consistent with this, *A. spiraecola* saliva-infiltrated plants with activated JA-dependent defenses disrupted the feeding behavior of *D. citri*, while *A. citricidus* saliva-infiltrated plants with suppressed JA-dependent defense enhanced the feeding efficiency of *D. citri*. This indicates that citrus aphids may differently affect subsequently infested *D. citri* by inducing and repressing JA signaling through injecting saliva during feeding. Determining the JA phytohormone content may help elucidate the role of JA in this interaction. On the other hand, SA is less effective in conferring resistance against piercing–sucking insects, including *D. citri* [26]. Some herbivorous insects can even activate SA to suppress the effective JA defense through hormone crosstalk [53]. In this study, while both citrus aphid species activated SA-dependent defenses, the overall effects on *D. citri* feeding behavior were different, suggesting that the SA pathways are less important when compared with the JA pathways.

## 5. Conclusions

In summary, we showed that saliva was involved in the contrasting plant-mediated effects of two citrus aphids on the feeding behavior of *D. citri* in *C. sinensis.* Moreover, two citrus aphids’ saliva differently modulated the expression of genes involved in plant SA and JA defense pathways, and this difference may explain this contrasting effect. This study provides new information regarding the function of saliva in plant-mediated inter-specific herbivore interactions. Further study will focus on identifying the salivary components, as well as elicitor screening. This work may help develop plant defense inducers and develop *D. citri*-resistant cultivars.

## Figures and Tables

**Table 1:** RT-qPCR primers for genes involved in SA and JA defense pathways.

Gene Name	Gene Function	Primer Sequence (5′-3′)
*CitGAPC1α*	Reference gene	F: ACTCCAGAGGGATGATGTGG
		R: ATGGGATCTCCTCTGGGTTC
*CitPAL*	SA biosynthesis	F: GGGGATCTGGTTCCTCTTTC
		R: CATAGAAGCCAGGCCAGAAC
*CitADT1*	SA biosynthesis	F: CACTCCTGTTGAGGACGACA
		R: CACCTGGTAAGCCCTGGTAA
*CitNPR1*	works downstream of SA	F: TGATAAGACCTTGCCACAACAC
		R: ACCGCAGGATTCAGATCTATGT
*CitPR1*	induced by SA	F: ACTGCAATCTTGTGCATTCG
		R: TTCACCCACAGTTTCACAGC
*CitPR2*	induced by SA	F: CCTTGTTCCCGCCATGAG
		R: GCCAAGAGCTCCAGTTTCGA
*CitAOS*	JA biosynthesis	F: GTTTCAGCTCGCTCCGTTAC
		R: GAGGTTGTGACACGCTTCCT
*CitAOC*	JA biosynthesis	F: GCGAGTGGGAATTACAGCAG
		R: TTAACCTGCCCACTCACTCC
*CitOPR3*	JA biosynthesis	F: ATGGTGCTGATTTGGTAGCC
		R: ACCCACTCAAAGGCGTGATA
*CitPI*	works downstream of JA	F: AATCTTCTCATCGCTTTATC
		R: TGCTTCGCACTTACAACT
*CitJMT*	methylation of jasmonate	F: GCCTGGACAAATACGCAAGAG
	into methyljasmonate	R: CGATTCTCCCACTGCCCTTA

## Data Availability

The data presented in this study are available upon request from the corresponding author.

## References

[1] Acevedo F.E., Rivera-Vega L.J., Chung S.H., Ray S., Felton G.W. (2015). Cues from chewing insects—The intersection of DAMPs, HAMPs, MAMPs and effectors. Curr. Opin. Plant Biol..

[2] Furstenberg-Hagg J., Zagrobelny M., Bak S. (2013). Plant defense against insect herbivores. Int. J. Mol. Sci..

[3] Thaler J.S., Humphrey P.T., Whiteman N.K. (2012). Evolution of jasmonate and salicylate signal crosstalk. Trends Plant Sci..

[4] Wasternack C., Feussner I. (2018). The oxylipin pathways: Biochemistry and function. Annu. Rev. Plant Biol..

[5] Jaouannet M., Rodriguez P.A., Thorpe P., Lenoir C.J.G., MacLeod R., Escudero-Martinez C., Bos J.I.B. (2014). Plant immunity in plant-aphid interactions. Front. Plant Sci..

[6] Howe G.A., Jander G. (2008). Plant immunity to insect herbivores. Annu. Rev. Plant Biol..

[7] Zhang P.-J., Li W.D., Huang F., Zhang J.M., Xu F.C., Lu Y.B. (2013). Feeding by whiteflies suppresses downstream jasmonic acid signaling by eliciting salicylic acid signaling. J. Chem. Ecol..

[8] Erb M., Reymond P. (2019). Molecular interactions between plants and insect herbivores. Annu. Rev. Plant Biol..

[9] Huang H.J., Zhang C.X., Hong X.Y. (2019). How does saliva function in planthopper–host interactions?. Arch. Insect Biochem. Physiol..

[10] Mondal H.A. (2020). Aphid saliva: A powerful recipe for modulating host resistance towards aphid clonal propagation. Arthropod-Plant Interact..

[11] Huang H.J., Ye Z.X., Lu G., Zhang C.X., Chen J.P., Li J.M. (2021). Identification of salivary proteins in the whitefly *Bemisia tabaci* by transcriptomic and LC–MS/MS analyses. Insect Sci..

[12] Su Y.-L., Li J.-M., Li M., Luan J.-B., Ye X.-D., Wang X.-W., Liu S.-S. (2012). Transcriptomic analysis of the salivary glands of an invasive whitefly. PLoS ONE.

[13] Wu Z.Z., Qu M.Q., Chen M.S., Lin J.T. (2021). Proteomic and transcriptomic analyses of saliva and salivary glands from the Asian citrus psyllid, *Diaphorina citri*. J. Proteom..

[14] Guo H., Zhang Y., Tong J., Ge P., Wang Q., Zhao Z., Zhu-Salzman K., Hogenhout S.A., Ge F., Sun Y. (2020). An aphid-secreted salivary protease activates plant defense in phloem. Curr. Biol..

[15] De Vos M., Jander G. (2009). *Myzus persicae* (green peach aphid) salivary components induce defence responses in *Arabidopsis thaliana*. Plant Cell Environ..

[16] Naessens E., Dubreuil G., Giordanengo P., Baron O.L., Minet-Kebdani N., Keller H., Coustau C. (2015). A secreted MIF cytokine enables aphid feeding and represses plant immune responses. Curr. Biol..

[17] Su Q., Peng Z., Tong H., Xie W., Wang S., Wu Q., Zhang J., Li C., Zhang Y. (2019). A salivary ferritin in the whitefly suppresses plant defenses and facilitates host exploitation. J. Exp. Bot..

[18] Elzinga D.A., De Vos M., Jander G. (2014). Suppression of plant defenses by a *Myzus persicae* (Green Peach Aphid) salivary effector protein. Mol. Plant Microbe Interact..

[19] Cui N., Lu H., Wang T., Zhang W., Kang L., Cui F. (2019). Armet, an aphid effector protein, induces pathogen resistance in plants by promoting the accumulation of salicylic acid. Philos. Trans. R. Soc. B.

[20] Ye W., Yu H., Jian Y., Zeng J., Ji R., Chen H., Lou Y. (2017). A salivary EF-hand calcium-binding protein of the brown planthopper *Nilaparvata lugens* functions as an effector for defense responses in rice. Sci. Rep..

[21] Grafton-Cardwell E.E., Stelinski L.L., Stansly P.A. (2013). Biology and Management of Asian Citrus Psyllid, Vector of the Huanglongbing Pathogens. Annu. Rev. Entomol..

[22] Liu X.Q., Jiang H.-B., Liu T.Y., Yang L., Fan J.Y., Xiong Y., Jing T.X., Lou B.H., Dou W., Wang J.J. (2020). A transcriptomic and proteomic analysis of the *Diaphorina citri* salivary glands reveals genes responding to *Candidatus* Liberibacter asiaticus. Front. Physiol..

[23] Yu X., Killiny N. (2018). The secreted salivary proteome of Asian citrus psyllid *Diaphorina citri*. Physiol. Entomol..

[24] Michaud J. (2002). Biological control of Asian citrus psyllid, *Diaphorina citri* (Hemiptera: Psyllidae) in Florida: A preliminary report. Entomol. News..

[25] Gao J., Xiu B., Sun Z., Arthurs S., Guo H., Gu S., Long J., Xia C., Hussain M., Mao R. (2022). *Aphis spiraecola* and *Aphis* (*Toxoptera*) *citricidus* differently manipulate plant metabolism to gain fitness in terms of population abundance or dispersal. Entomol. Exp. Et Appl..

[26] Gao J., Tao T., Arthurs S.P., Ye F., An X., Hussain M., Mao R. (2023). Plant jasmonic acid mediated contrasting effects of two citrus aphid species on *Diaphorina citri* Kuwayama. Pest Manag. Sci..

[27] Capinera J.L. (2000). Melon Aphid or Cotton Aphid, Aphis gossypii Glover (Insecta: Hemiptera: Aphididae).

[28] McCoy C.W., Samson R.A., Boucias D.G., Osborne L.S., Pena J.E., Buss L.J. (2009). Pathogens Infecting Insects and Mites of Citrus.

[29] Zhang Y., Fan J., Francis F., Chen J.L. (2017). Watery saliva secreted by the grain aphid Sitobion avenae stimulates aphid resistance in wheat. J. Agric. Food Chem..

[30] Francis M.I., Kostenyuk I., Orbović V., Loskutov A., Zolotukhin M., Graham J.H. (2011). Automated needle-free injection method for delivery of bacterial suspensions into citrus leaf tissues. J. Phytopathol..

[31] Ebert T.A., Backus E.A., Cid M., Fereres A., Rogers M.E. (2015). A new SAS program for behavioral analysis of electrical penetration graph data. Comput. Electron. Agric..

[32] Miranda M.P., Yamamoto P.T., Garcia R.B., Lopes J.P.A., Lopes J.R.S. (2016). Thiamethoxam and imidacloprid drench applications on sweet orange nursery trees disrupt the feeding and settling behaviour of *Diaphorina citri* (Hemiptera: Liviidae). Pest Manag. Sci..

[33] Carmo-Sousa M., Garcia R.B., Wulff N.A., Fereres A., Miranda M.P. (2020). Drench application of systemic insecticides disrupts probing behavior of *Diaphorina citri* (Hemiptera: Liviidae) and inoculation of *Candidatus* Liberibacter asiaticus. Insects.

[34] Nehela Y., Hijaz F., Elzaawely A.A., El-Zahaby H.M., Killiny N. (2018). Citrus phytohormonal response to *Candidatus* Liberibacter asiaticus and its vector *Diaphorina citri*. Physiol. Mol. Plant Pathol..

[35] Ibanez F., Suh J.H., Wang Y., Stelinski L.L. (2019). Long-term, sustained feeding by Asian citrus psyllid disrupts salicylic acid homeostasis in sweet orange. BMC Plant Biolog..

[36] Oliveira Coqueiro D.S., de Souza A.A., Takita M.A., Rodrigues C.M., Kishi L.T., Machado M.A. (2015). Transcriptional profile of sweet orange in response to chitosan and salicylic acid. BMC Genom..

[37] Zhao W., Baldwin E.A., Bai J., Plotto A., Irey M. (2019). Comparative analysis of the transcriptomes of the calyx abscission zone of sweet orange insights into the huanglongbing-associated fruit abscission. Hortic. Res..

[38] Ohgushi T. (2005). Indirect interaction webs: Herbivore-induced effects through trait change in plants. Annu. Rev. Ecol. Evol. Syst..

[39] Hu X.S., Liu X.F., Zhao H.Y. (2006). Development and application of electrical penetration graph (EPG) technique. Plant Prot..

[40] Liu B.M., Yan F.M., Chu D., Pan H.P., Jiao X.G., Xie W., Wu Q.J., Wang S.L., Xu B.Y., Zhou X.G. (2012). Difference in feeding behaviors of two invasive whiteflies on host plants with different suitability: Implication for competitive displacement. Int. J. Biol. Sci..

[41] Nowak H., Komor E. (2010). How aphids decide what is good for them: Experiments to test aphid feeding behaviour on *Tanacetum vulgare* (L. ) using different nitrogen regimes. Oecologia.

[42] Zhang Y., Fu Y., Francis F., Liu X., Chen J. (2021). Insight into watery saliva proteomes of the grain aphid, *Sitobion avenae*. Arch. Insect Biochem. Physiol..

[43] Acevedo F.E., Smith P., Peiffer M., Helms A., Tooker J., Felton G.W. (2019). Phytohormones in fall armyworm saliva modulate defense responses in plants. J. Chem. Ecol..

[44] Alborn H.T., Hansen T.V., Jones T.H., Bennett D.C., Tumlinson J.H., Schmelz E.A., Teal P.E. (2007). Disulfooxy fatty acids from the American bird grasshopper *Schistocerca americana*, elicitors of plant volatiles. Proc. Natl. Acad. Sci. USA.

[45] Chen Y., Singh A., Kaithakottil G.G., Mathers T.C., Gravino M., Mugford S.T., van Oosterhout C., Swarbreck D., Hogenhout S.A. (2020). An aphid RNA transcript migrates systemically within plants and is a virulence factor. Proc. Natl. Acad. Sci. USA.

[46] Yan Y., Zhang H., Yang Y., Zhang Y., Guo J., Liu W., Wan F. (2016). Plant defense responses induced by *Bemisia tabaci* Middle East—Asia Minor 1 salivary components. Entomol. Exp. Et Appl..

[47] Ali J.G., Agrawal A.A. (2012). Specialist versus generalist insect herbivores and plant defense. Trends Plant Sci..

[48] Mostefaoui H., Allal-Benfekih L., Djazouli Z.E., Petit D., Saladin G. (2014). Why the aphid *Aphis spiraecola* is more abundant on clementine tree than *Aphis gossypii*?. Comptes Rendus Biol..

[49] Brlansky R., Roy A., Damsteegt V. (2011). Stem-pitting Citrus tristeza virus predominantly transmitted by the brown citrus aphid from mixed infections containing non-stem-pitting and stem-pitting isolates. Plant Dis..

[50] Wang L., Wu J. (2013). The essential role of jasmonic acid in plant-herbivore interactions—Using the wild tobacco *Nicotiana attenuata* as a model. J. Genet. Genom..

[51] Cao H.H., Wang S.H., Liu T.X. (2014). Jasmonate-and salicylate-induced defenses in wheat affect host preference and probing behavior but not performance of the grain aphid, *Sitobion avenae*. Insect Sci..

[52] Zarate S.I., Kempema L.A., Walling L.L. (2007). Silverleaf whitefly induces salicylic acid defenses and suppresses effectual jasmonic acid defenses. Plant Physiol..

[53] Zhu-Salzman K., Bi J.L., Liu T.X. (2005). Molecular strategies of plant defense and insect counter-defense. Insect Sci..

